# Distribution and Molecular Characterization of Human Adenovirus and Epstein-Barr Virus Infections in Tonsillar Lymphocytes Isolated from Patients Diagnosed with Tonsillar Diseases

**DOI:** 10.1371/journal.pone.0154814

**Published:** 2016-05-02

**Authors:** Farzaneh Assadian, Karl Sandström, Kåre Bondeson, Göran Laurell, Adnan Lidian, Catharina Svensson, Göran Akusjärvi, Anders Bergqvist, Tanel Punga

**Affiliations:** 1 Department of Medical Biochemistry and Microbiology, Uppsala Biomedical Center, Uppsala University, Uppsala, Sweden; 2 Department of Surgical Sciences, Otorhinolaryngology and Head and Neck Surgery, Uppsala University, Uppsala, Sweden; 3 Department of Medical Sciences, Clinical Microbiology and Infectious Medicine, Uppsala University, Uppsala, Sweden; University of St Andrews, UNITED KINGDOM

## Abstract

Surgically removed palatine tonsils provide a conveniently accessible source of T and B lymphocytes to study the interplay between foreign pathogens and the host immune system. In this study we have characterised the distribution of human adenovirus (HAdV), Epstein-Barr virus (EBV) and human cytomegalovirus (HCMV) in purified tonsillar T and B cell-enriched fractions isolated from three patient age groups diagnosed with tonsillar hypertrophy and chronic/recurrent tonsillitis. HAdV DNA was detected in 93 out of 111 patients (84%), while EBV DNA was detected in 58 patients (52%). The most abundant adenovirus type was HAdV-5 (68%). None of the patients were positive for HCMV. Furthermore, 43 patients (39%) showed a co-infection of HAdV and EBV. The majority of young patients diagnosed with tonsillar hypertrophy were positive for HAdV, whereas all adult patients diagnosed with chronic/recurrent tonsillitis were positive for either HAdV or EBV. Most of the tonsils from patients diagnosed with either tonsillar hypertrophy or chronic/recurrent tonsillitis showed a higher HAdV DNA copy number in T compared to B cell-enriched fraction. Interestingly, in the majority of the tonsils from patients with chronic/recurrent tonsillitis HAdV DNA was detected in T cells only, whereas hypertrophic tonsils demonstrated HAdV DNA in both T and B cell-enriched fractions. In contrast, the majority of EBV positive tonsils revealed a preference for EBV DNA accumulation in the B cell-enriched fraction compared to T cell fraction irrespective of the patients' age.

## Introduction

Tonsils are collections of incompletely encapsulated lymphoid tissues located beneath, but in contact with the epithelium of the upper aero-digestive tract [1]. The paired palatine tonsils together with the nasopharyngeal tonsils (adenoids) and lingual tonsils form prominent secondary lymphoepithelial tissues responsible for the initiation of the immune responses against inhaled or ingested pathogens. Histologically, the palatine tonsils contain numerous lymphoid follicles with germinal centers, which are the sites for B cell maturation and differentiation (B-cell areas). The palatine tonsils also accommodate T cells, which are mainly located in the extrafollicular regions (T-cell areas) [1–3].

Tonsillar hypertrophy and chronic/recurrent tonsillitis are common tonsillar diseases, usually caused by viral or bacterial pathogens [4, 5]. Children with tonsillar hypertrophy commonly display complications like nasal obstruction, obstructive sleep apnea, snoring, and may even experience psychosocial problems [6]. These complications can be treated by complete or partial removal of the tonsils, known as tonsillectomy and tonsillotomy, respectively [6, 7].

The major viral agents of tonsillar inflammation are adenovirus, parainfluenza virus, rhinovirus, influenza virus, respiratory syncytial virus and the herpes family of viruses [Epstein-Barr virus (EBV) and human cytomegalovirus (HCMV)] [4, 5, 8, 9]. Human adenoviruses (HAdVs) are classified into seven distinct species, A to G, with at least 70 types described thus far [10–12]. HAdVs cause a broad spectrum of clinical diseases, such as respiratory tract infections, epidemic keratoconjunctivitis and gastroenteritis [11, 13–15]. Viruses in species B (HAdV-3, 7, 11, 14, 16, 21, 34, 35, 50, 55, 66 and 68), C (HAdV-1, 2, 5, 6 and 57) and E (HAdV-4) are typically connected to infections of the respiratory tract [16].

The pathogenesis caused by HAdVs can be due to short-term lytic or long-term latent/persistent infections. During the lytic replicative cycle in epithelial cells, the recipient cell is efficiently lysed within a few days post-infection, whereas in non-lytic, long-term latent/persistent infections the virus survives in a dormant state in lymphoid cells [9, 17, 18]. Several studies have shown the presence of HAdVs in the palatine tonsils and adenoids [4, 9, 19, 20]. In fact, HAdV was first identified as a cytopathogenic agent from human adenoids [17]. Recent virus typing analyses have demonstrated that viral DNA, particularly from species C viruses (HAdV-1, HAdV-2 and HAdV-5), is often detectable in T lymphocytes of surgically removed tonsils [9, 21, 22]. This observation suggests that tonsillar T lymphocytes might serve as the reservoir for latent HAdV in infected individuals.

EBV is an orally transmitted γ-1 herpesvirus, which infects most individuals in childhood or early adulthood [23]. Palatine tonsils are the initial sites used by EBV to invade the host and may therefore serve as the reservoir for EBV infection [24, 25]. In the tonsils, EBV is capable of infecting not only B lymphocytes and lymphoepithelial cells, but also T lymphocytes [26–28].

HCMV is a β herpesvirus, which is ubiquitously spread in the human population. HCMV infects a wide variety of the cells within the host [11, 29]. Monocytes are known to be the major site of HCMV latent infection in peripheral blood mononuclear cells [30]. However, the role of tonsils as the sites of HCMV latency has not been established.

In order to investigate the prevalence of HAdV, EBV and HCMV in patients with tonsillar hypertrophy and chronic/recurrent tonsillitis, we have performed a study on isolated tonsillar B and T lymphocytes, obtained from patients undergoing tonsillectomy or tonsillotomy. Our results show that HAdV predominantly resides in tonsillar T lymphocytes. In contrast to HAdV, EBV was mainly detected in the tonsillar B lymphocytes. None of the patients were tested positive for HCMV.

## Materials and Methods

### Clinical specimens

This study was carried out from March 2014 to March 2015. This project was approved by the Uppsala Ethical Review Board (Dnr. 2013/387/2). Prior to participation in the study, all patients or guardians signed the informed consent form. The left and right palatine tonsils were obtained from 111 nonconsecutive patients, diagnosed with symptomatic tonsillar hypertrophy or chronic/recurrent tonsillitis and undergoing routine tonsillectomy or tonsillotomy at Uppsala University Hospital, Sweden. Pairs of tonsils from individual donors were analysed separately. Patients were categorized according to the size of palatine tonsils and history of prior tonsillar infections.

55 patients (age 1 to 58) were diagnosed with tonsillar hypertrophy. These patients had enlarged tonsils with a history of obstructive sleep apnea. The patients did not have any history of recurrent tonsillitis.

56 patients (age 2 to 42) were diagnosed with chronic/recurrent tonsillitis, generally according to the Paradise guidelines, which means the patients had at least seven episodes of sore throat in the previous year, at least five episodes per year in the previous two years, or at least three episodes in each of the previous three years.

### Isolation of tonsillar B and T lymphocytes

Fresh surgically removed tonsils were processed into single cell suspensions as previously described [21]. Briefly, tissue was cut into 3–10 mm fragments and smoothly squeezed through the cell strainer (pore size of 100 μm) using the plunger end of a plastic syringe. Viable mononuclear cells (MNCs) were purified by density gradient centrifugation. CD3^+^ T lymphocytes were positively selected and isolated from the tonsillar MNCs using the Magnetic Activated Cell Sorting method (Miltenyi Biotech, Lund, Sweden), as previously described [21]. Briefly, 3 × 10^7^ MNCs were washed in ice-cold separation buffer [PBS (pH 7.2), 0.5% bovine serum albumin (BSA) and 2 mM EDTA] and afterwards incubated with CD3 antibody coupled to the magnetic beads (Miltenyi Biotech, Lund, Sweden). After appropriate incubation time the cell suspension was washed in the separation buffer, loaded onto the separation column and subjected to the magnetic field for the positive selection of the CD3^+^ cells. The flow-through fraction contained the CD3^-^ cell population, which was considered as the B cell-enriched fraction (with purity of 92%). The magnetically retained CD3^+^ cells were eluted from the column and considered as the T cell-enriched fraction (with purity of 97%). Enrichment of tonsillar B and T lymphocyte fractions was confirmed by flow cytometry [21].

### DNA extraction

DNA was extracted from the B and T cell-enriched fractions using the NucleoSpin Blood kit (Macherey-Nagel, Düren, Germany) according to the manufacturer's instructions.

### Viral DNA quantification

Extracted DNA from B and T lymphocyte-enriched fractions were analysed using the previously described quantitative TaqMan real time PCR (qPCR) procedures set up to detect HAdV [31], EBV [32], and HCMV [33]. All assays were carried out in a Corbett Rotor-Gene 3000 thermo cycler (Corbett Research, Australia). The qPCR reactions were performed with TaqMan Universal PCR Master Mix (Thermo Fischer, Waltham, MA, USA) using approximately 250 ng of template DNA in a final reaction volume of 25 μL. The thermocycling profile was 1 cycle of 95°C for 15 min followed by 50 cycles of 95°C for 15 sec and 57°C for 60 sec. Primers and probes were synthesized by Eurogentec (Seraing, Belgium), ThermoFisher Scientific (Waltham, MA) and SGS DNA (Köping, Sweden). As external calibrators, normalized HAdV-5 DNA (Advanced Biotechnologies Inc., Columbia, MD) and DNA extracted from Namalwa cells containing two EBV copies per genome were used [34]. Quantitation was performed using imported standard curves based on previously determined PCR efficiency and adjustment against a reference point analysed in the same run. Proficiencies of the assays were verified using external quality assessment programs from Instand (www.instandev.de; HAdV) and Quality Control for Molecular Diagnostics (www.qcmd.org; HAdV, EBV and HCMV). Adjustment for cell number was performed by analysing each sample for the cellular DNA content by performing qPCR for the RNaseP as an internal control following the CDC rRTPCR protocol [35].

### Nested PCR

To genotype the HAdV in the enriched B and T lymphocyte fractions, touchdown nested PCR was performed to amplify the highly variable regions (HVR1-6) of the hexon gene. The external primers were AdHVR-F1 (5´-ACAGGAYGCYTCGGRGTAYCT-3´) and AdHVR-R1 (5´-CAGTTCDGTRTTTCTGTCYTGCA-3´) and the internal primers were AdHVR-F2 (5´-CCCTACTCCGGYACNGCYTAYAA-3´) and AdHVR-R2 (5´-ACTCCCATRTTDCCHGTRCWGTT-3´). The AdHVR-F1 and AdHVR-R1 external primers are respectively corresponding to positions 18888–18908 and 19955–19977 in the HAdV-2 genome (GenBank AC_000007), while internal primers (AdHVR-F2 and AdHVR-R2) are corresponding to nucleotides 19179–19202 and 19891–19913, respectively. The expected size of the PCR product obtained from the first and second round of amplification was 1089 and 734 nucleotides, respectively. This assay detects most HAdV genotypes [36, 37]. The PCR amplifications were carried out in a 25 μL reaction mixtures consisting of 10× *Taq* buffer, 1.5 mM MgCl_2_, 0.2 mM of each deoxynucleotide triphosphate, 1.25 U of *Taq* DNA polymerase (all from Thermo Scientific, Stockholm, Sweden) and 0.8 μM each primer. The first round amplification was performed on 5 μL of extracted DNA with the concentration of 50 ng/μL, while 5 μL of first round PCR product was used as a template for the second round amplification. Every PCR run included a blank control (ultrapure water), a negative control (DNA extracted from non-infected BJAB cells) and a positive control (DNA extracted from HAdV-5 infected BJAB cells) [18]. The touchdown PCR amplification program was carried out as 1 cycle at 95°C for 4 min, 25 cycles of 94°C for 35 sec, 58°C for 35 sec (-0.2/cycle) and 72°C for 1 min, followed by 20 cycles of 94°C for 35 sec, 53°C for 35 sec and 72°C for 1 min and a final elongation of 5 min at 72°C.

### Sequence Analysis

PCR amplicons were purified using NucleoSpin gel and PCR clean-up kit (Macherey-Nagel, Düren, Germany) and sequenced unidirectionally using the Sanger sequencing method. The CLC Sequence Viewer 7.6 (CLC bio A/S, Aarhus, Denmark) was used to view and edit the raw sequencing data. Typing was verified by identification of homologous nucleotide sequences in the GenBank database, using the BLASTn program (National Center for Biotechnology Information, Bethesda, MD, USA), where identities over 90 percent were considered significant.

### Statistical analysis

Data were plotted and statistically analysed using GraphPad Prism 6 (GraphPad Software, San Diego, CA, USA). Chi-square and Fisher's exact tests were used to examine seasonal variation and prevalence of HAdV and EBV in tonsillar lymphocytes. Unpaired *t*-test, paired *t*-test, Mann-Whitney U test, Fisher's exact test and one-way ANOVA were applied to compare the groups regarding HAdV and EBV copy number or T/B ratio of HAdV copy numbers. Statistical significance: *p<0.05, ** p<0.01, *** p<0.001, **** p<0.0001.

## Results

### Clinical profile

Left and right tonsil samples were obtained from a cohort of 111 Swedish patients who underwent tonsillectomy or tonsillotomy due to diagnosed tonsillar hypertrophy (55 patients) or chronic/recurrent tonsillitis (56 patients). The patients were further categorized into three age groups: 1–9, 10–19 and ≥20 years. A significant difference was observed between the gender ratio in tonsillar hypertrophy compared to chronic/recurrent tonsillitis group. The chronic/recurrent tonsillitis group consisted of more female than male patients, whereas more male patients were found in the cohort with tonsillar hypertrophy (Table 1, p<0.01). Most of the young patients (1–9 years) showed signs of tonsillar hypertrophy, while in the adolescent and adult patient groups (10–19 and ≥20 years) the pattern of the tonsillar disease changed towards the chronic/recurrent tonsillitis (Table 1).

**Table 1:** Characteristics of the study population.

Age	Number of patients	Tonsillar hypertrophy^a^		Chronic/recurrent tonsillitis^a^	
		Female	Male	Female	Male
**1–9**	47	16 (34%)	26 (55%)	4 (9%)	1 (2%)
**10–19**	26	3 (11%)	2 (8%)	12 (46%)	9 (35%)
**≥ 20**	38	3 (8%)	5 (13%)	21 (55%)	9 (24%)
**Total**	111	22 (20%)	33 (30%)	37 (33%)	19 (17%)

^a^ Percentage of the patients in the respective age groups

### Detection of HAdV, EBV and HCMV DNA in tonsillar lymphocytes

To assess the prevalence of HAdV, EBV and HCMV in human palatine tonsils, tonsillar material from tonsillectomy or tonsillotomy surgeries were analysed. Tonsillar B and T lymphocytes were isolated from tonsillar MNCs, obtained from fragmented palatine tonsil tissue, by using a CD3^+^ antibody-based immunopurification. Using this isolation approach we obtained enriched B and T lymphocyte fractions, which were more than 90% pure, as measured by flow cytometry [21]. Total DNA was extracted from the enriched tonsillar B and T lymphocyte fractions and viral DNA was quantified by qPCR using virus-specific primers. The individual viral DNA copy number in B and T lymphocytes were summarised to obtain the viral DNA copy number in tonsillar lymphocytes. HAdV DNA was detected in 93 out of 111 patients (84%, Table 2), while EBV DNA was detected in 58 patients out of 111 patients (52%, Table 3). None of the patients were tested positive for HCMV. Of all the patients, 43 (39%) showed a co-infection of HAdV and EBV (Table 4). Notably, no double-negative samples were observed in the adult patient group (≥20 years) diagnosed with tonsillar hypertrophy or chronic/recurrent tonsillitis (Table 4, p<0.05).

**Table 2:** Prevalence of HAdV DNA in patients diagnosed with tonsillar hypertrophy and chronic/recurrent tonsillitis.

	HAdV positive patients^a^					
Age		Tonsillar hypertrophy^a^		Chronic/recurrent tonsillitis^a^		HAdV negative patients^b^
		Female	Male	Female	Male	
**1–9**	44/47 (94%)	15/16 (94%)	25/26 (96%)	3/4 (75%)	1/1 (100%)	3/47 (6%)
**10–19**	21/26 (81%)	2/3 (67%)	1/2 (50%)	11/12 (92%)	7/9 (78%)	5/26 (19%)
**≥ 20**	28/38 (74%)	2/3 (67%)	4/5 (80%)	16/21 (76%)	6/9 (67%)	10/38 (26%)
**Total**	93/111 (84%)	19/22 (86%)	30/33 (91%)	30/37 (81%)	14/19 (74%)	18/111 (16%)

^a^ Percentage of the HAdV positive patients in the respective age groups

^b^ Percentage of the HAdV negative patients in the respective age groups

**Table 3:** Prevalence of EBV DNA in patients diagnosed with tonsillar hypertrophy and chronic/recurrent tonsillitis.

	EBV positive patients^a^					
Age		Tonsillar hypertrophy^a^		Chronic/recurrent tonsillitis^a^		EBV negative patients^b^
		Female	Male	Female	Male	
**1–9**	15/47 (32%)	6/16 (37.5%)	8/26 (31%)	0/4 (0%)	1/1 (100%)	32/47 (68%)
**10–19**	17/26 (65%)	0/3 (0%)	2/2 (100%)	9/12 (75%)	6/9 (67%)	9/26 (35%)
**≥ 20**	26/38 (68%)	2/3 (67%)	4/5 (80%)	15/21 (71%)	5/9 (56%)	12/38 (32%)
**Total**	58/111 (52%)	8/22 (36%)	14/33 (42%)	24/37 (80%)	12/19 (63%)	53/111 (48%)

^a^ Percentage of the EBV positive patients in the respective age groups

^b^ Percentage of the EBV negative patients in the respective age groups

**Table 4:** Prevalence HAdV and EBV co-infection in patients diagnosed with tonsillar hypertrophy and chronic/recurrent tonsillitis.

	HAdV/EBV double-positive patients^a^					
Age		Tonsillar hypertrophy^a^		Chronic/recurrent tonsillitis^a^		HAdV/ EBV double-negative patients^b^
		Female	Male	Female	Male	
**1–9**	13/47 (28%)	5/16 (31%)	7/26 (27%)	0/4 (0%)	1/1 (100%)	1/47 (0.5%)
**10–19**	14/26 (54%)	0/3 (0%)	1/2 (50%)	8/12 (67%)	5/9 (56%)	2/26 (8%)
**≥ 20**	16/38 (42%)	1/3 (33%)	3/5 (60%)	10/21 (48%)	2/9 (22%)	0/38 (0%)
**Total**	43/111 (39%)	6/22 (27%)	11/33 (33%)	18/37 (49%)	8/19 (42%)	3/111 (1%)

^a^ Percentage of the HAdV/EBV double-positive patients in the respective age groups

^b^ Percentage of the HAdV/EBV double-negative patients in the respective age groups

The prevalence of HAdV was significantly higher in the young patients (1–9 years) compared to adult patients (Table 2, p<0.05). In contrast, the prevalence of EBV was significantly lower in the young patients compared to the adolescent and adult patients (Table 3, p<0.05 and p <0.01, respectively). For both HAdV and EBV (Tables 2 and 3) most of the young patients (1–9 years) showed signs of tonsillar hypertrophy, while in the adolescent and adult patient groups (10–19 and ≥20 years) the pattern of the tonsillar disease shifted towards the chronic/recurrent tonsillitis.

**Fig 1:**
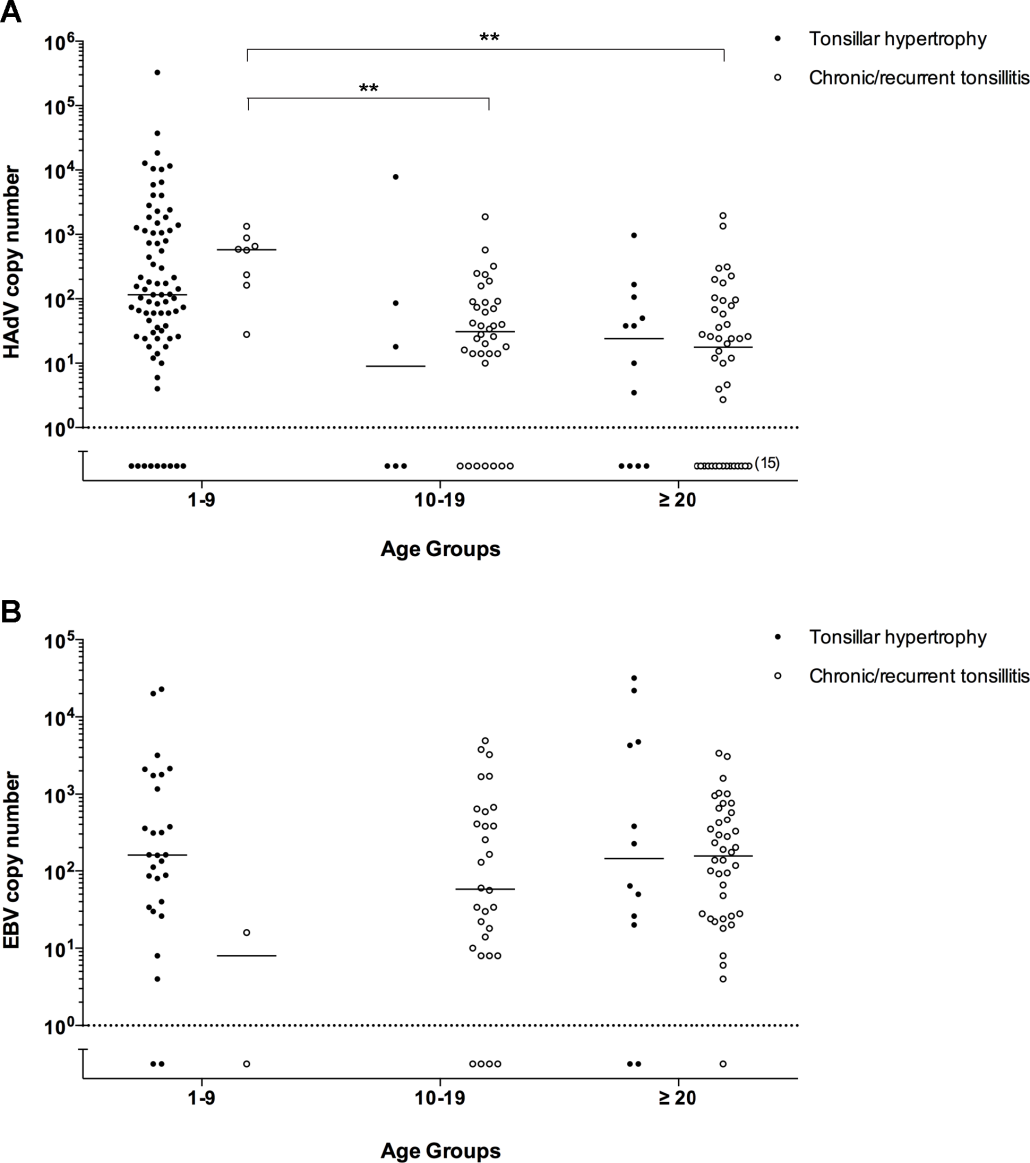
Viral DNA titers in the palatine tonsils from patients diagnosed with tonsillar hypertrophy and chronic/recurrent tonsillitis. HAdV (A) and EBV (B) DNA copy number (log10/10^6^ cells) is shown as the sum of the virus DNA copy numbers in the isolated T and B cell-enriched populations from each tonsil. Each data point indicates a patient´s single tonsil (left or right) sample. Age groups (1–9 years, 10–19 years and ≥20 years) are shown on x-axis. Dotted line represents the threshold below which virus DNA was undetectable. The horizontal bar shows median value for each group. ** p<0.01, determined by one-way ANOVA test.

The HAdV DNA copy number in individual tonsils ranged from 2.8 × 10^0^ to 3.2 × 10^5^ copies per 10^6^ tonsillar lymphocytes (Fig 1A). In 38 patients out of 93 (41%), HAdV DNA was detected only in one of the tonsils. Within HAdV positive patients, young patients diagnosed with chronic/recurrent tonsillitis had significantly higher HAdV copy number compared to adolescent and adult patients with the same diagnosis. No significant HAdV DNA copy number difference was observed between patients diagnosed with tonsillar hypertrophy and chronic/recurrent tonsillitis in each studied age group (Fig 1A).

In EBV positive tonsils, the DNA copy number varied from 4 × 10^0^ to 3.2 × 10^4^ copies per 10^6^ tonsillar lymphocytes. In 10 patients out of 58 (17%), EBV DNA was detected only in one of the tonsils. No significant difference was observed between the EBV DNA copy number in patients from different age groups or diagnosis (Fig 1B).

### Seasonal variation of HAdV and EBV infections in tonsillar lymphocytes

**Fig 2:**
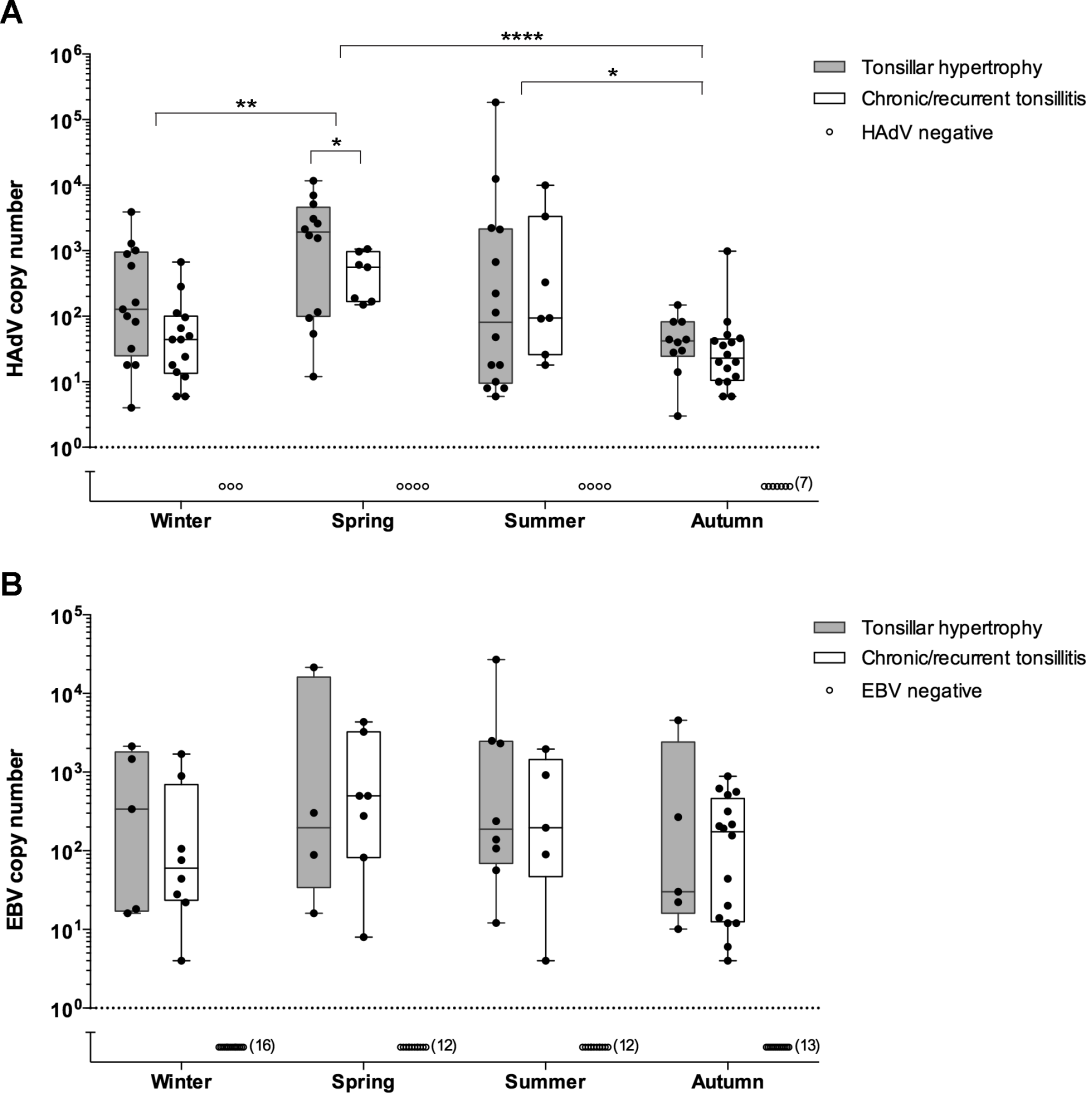
Seasonal detection of HAdV and EBV DNA in patients diagnosed with tonsillar hypertrophy and chronic/recurrent tonsillitis. HAdV (A) and EBV (B) DNA copy numbers in patients diagnosed with tonsillar hypertrophy and chronic/recurrent tonsillitis are shown. Each data point represents the respective viral copy number (log10/10^6^ cells) and indicates the sum of the virus titers in each patient´s left and right tonsil samples. Winter: Dec, Jan, Feb, Spring: Mar, Apr, May, Summer: Jun, Jul, Aug, Autumn: Sep, Oct, Nov. Dotted line represents the threshold below which virus DNA was undetectable. The line in the middle of the box is plotted at the median. *p<0.05, ** p<0.01, **** p<0.0001, determined by one-way ANOVA or unpaired *t*-test.

Previous studies have demonstrated that HAdV infections show seasonal variations [38]. To assess the seasonal variation of HAdV and EBV infections, the summarised viral DNA copy numbers from enriched tonsillar B and T lymphocyte fractions were analysed. In this study, seasonality was not observed in the incidence of HAdV or EBV infections, although a seasonal variation in the HAdV infection appears to exist in positive patients. Patients who underwent surgery in the spring (March, April and May) demonstrated the highest HAdV copy number. In these patients the HAdV DNA copy number was significantly higher compared to the patients who underwent surgery in winter (December, January and February) and autumn (September, October and November) (Fig 2A). A seasonal variation was, however, not observed for EBV (Fig 2B).

### Accumulation of HAdV and EBV DNA in enriched tonsillar B and T lymphocyte fractions

**Fig 3:**
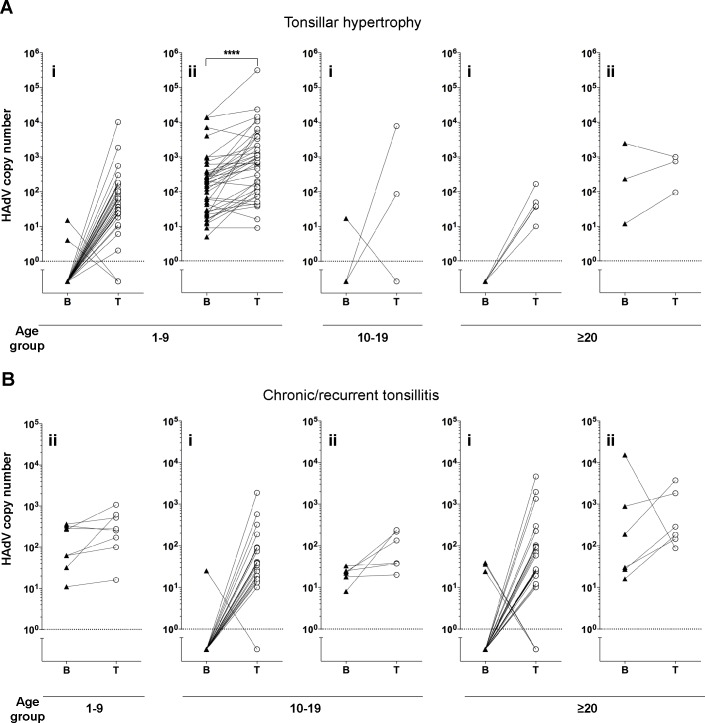
Accumulation of HAdV DNA in tonsillar B and T cell-enriched fractions obtained from infected tonsils. HAdV is predominantly detected in tonsillar T lymphocytes. HAdV DNA copy number is shown in tonsillar B and T cell-enriched fractions in patients with tonsillar hypertrophy (A) and chronic/recurrent tonsillitis (B). Tonsils infected in either B or T cells (i) and tonsils infected in both B and T cells (ii) are shown. Triangles and circles represent the HAdV DNA copy numbers (log10/10^6^ cells) in enriched B and T lymphocyte fractions. Each line connects the B and T cell data points belonging to a single tonsil sample. Dotted line represents the threshold below which virus DNA was undetectable. **** p<0.0001, determined by paired *t*-test.

**Fig 4:**
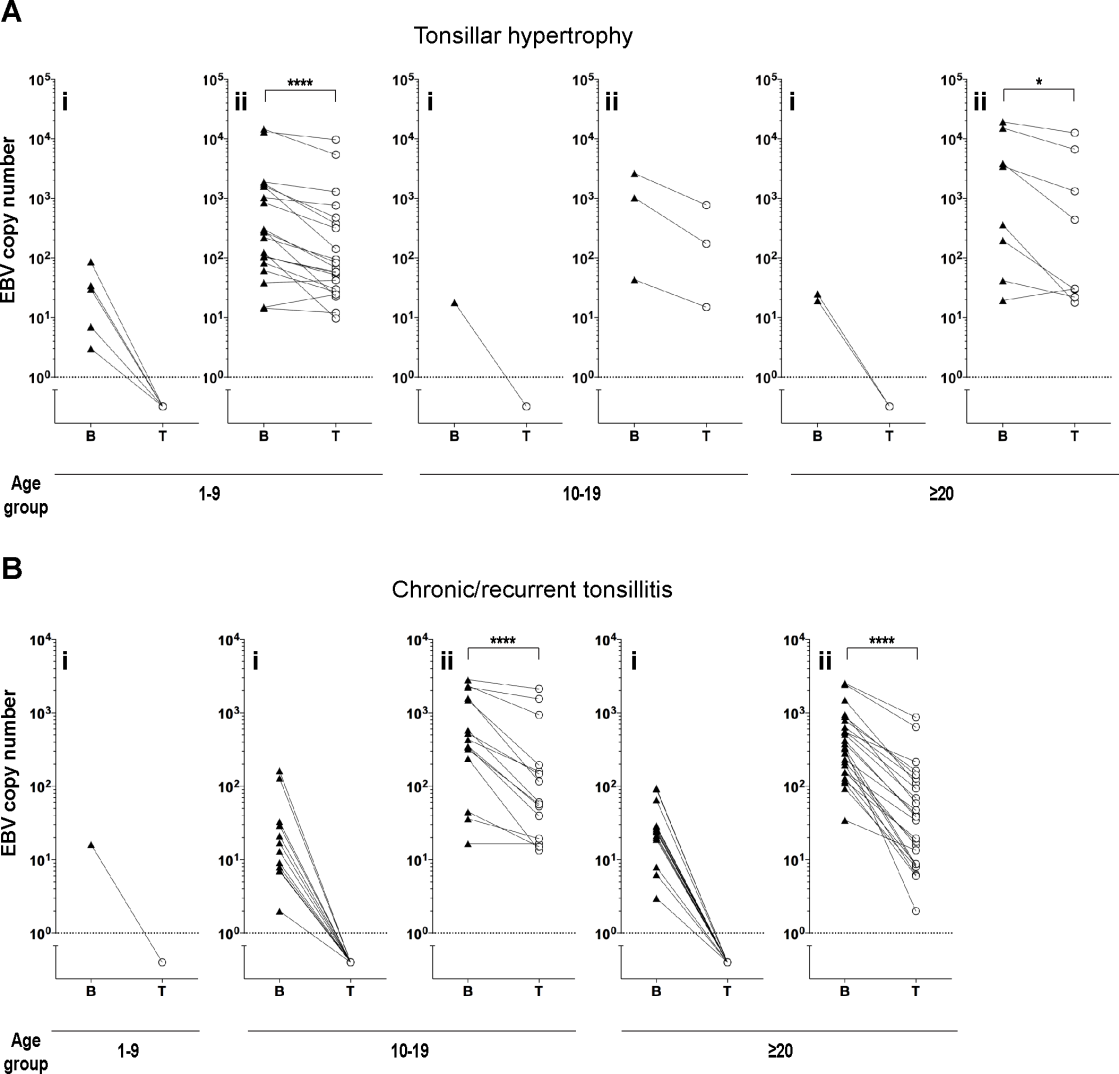
Accumulation of EBV DNA in tonsillar B and T cell-enriched fractions obtained from infected tonsils. EBV DNA is predominantly detected in tonsillar B cell-enriched fraction. EBV DNA copy number is shown in tonsillar B and T cell-enriched fractions in patients with tonsillar hypertrophy (A) and chronic/recurrent tonsillitis (B). Tonsils infected in either B or T cells (i) and tonsils infected in both B and T cells (ii) are shown. Triangles and circles represent the EBV DNA copy numbers (log10/10^6^ cells) in enriched B and T lymphocyte fractions. Each line connects the B and T cell data points belonging to a single tonsil sample. Dotted line represents the threshold below which virus DNA was undetectable. *p<0.05, **** p<0.0001, determined by paired *t*-test.

To determine the tonsillar lymphocyte subpopulation harboring HAdV and EBV, the viral DNA copy number from enriched B and T cell fractions was calculated for each tonsil sample and presented as samples containing viral DNA in either B or T cells or in both cell types (Figs 3 and 4). The majority (89%) of the tonsils from patients diagnosed with either tonsillar hypertrophy or chronic/recurrent tonsillitis, showed a preferred accumulation of HAdV DNA in T cells (Fig 3). The highest calculated HAdV DNA copy number was 3.1 × 10^5^ per 10^6^ T lymphocytes. Young patients (1–9 years) diagnosed with tonsillar hypertrophy showed significantly higher HAdV DNA copy number in T cells compared to B cell-enriched fraction (Fig 3Aii). However, in a few tonsil samples (11%), the HAdV DNA copy number was equal in the B and T cell-enriched fractions or even higher in the B cell-enriched fraction (Fig 3). Compared to the young patient group, the adolescent and adult patient groups demonstrated a significantly higher number of the tonsil samples, with HAdV DNA only in the T lymphocyte-enriched fractions (Fisher's exact test, p<0.0001). Similarly, in comparison to the tonsillar hypertrophy group, the patients diagnosed with recurrent/chronic tonsillitis showed significantly higher number of the tonsil samples, where HAdV DNA was only detected in the T lymphocyte-enriched fractions (Fisher's exact test, p<0.01). To further verify HAdV DNA accumulation in tonsillar lymphocytes, B and T cells were isolated from three patients' MNCs using the fluorescence-activated cell sorting (FACS) method (S1A Fig). Both, highly pure, CD20^+^-immunosorted (B cells) and CD2^+^-immunosorted (T cells) tonsillar lymphocyte fractions showed clear presence of HAdV DNA (S1B Fig).

**Fig 5:**
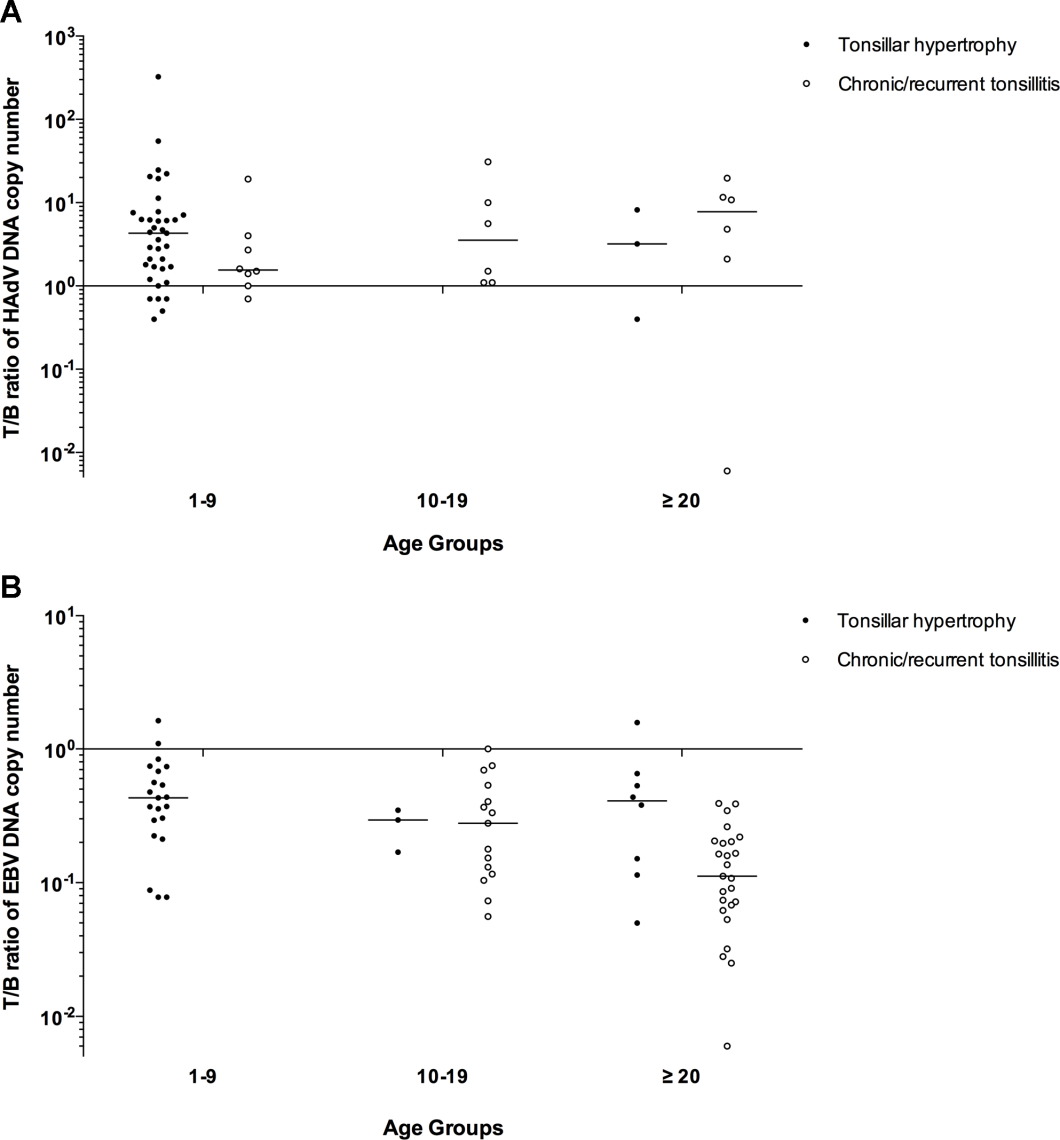
HAdV and EBV DNA copy number in infected tonsils. Each data point indicates the ratio (log10) of HAdV (A) and EBV (B) DNA copy number in T cells to the virus DNA copy number in B cell-enriched fraction (T/B ratio). Age groups (1–9 years, 10–19 years and ≥20 years) are shown on x-axis. The horizontal bar shows median value for each group.

In the majority (93%) of EBV positive tonsils the analysis of the EBV DNA copy number revealed a preference for EBV DNA accumulation in the B cell-enriched fraction compared to the T cell population (Fig 4). The patients diagnosed with tonsillar hypertrophy (1–9 years and ≥20 years) and chronic tonsillitis (10–19 years and ≥20 years) showed significantly higher EBV DNA copy number in the B cell-enriched fraction compared to the T cell fraction (Fig 4). Compared to the tonsillar hypertrophy group, the patients diagnosed with recurrent/chronic tonsillitis showed significantly higher number of the tonsil samples, where EBV DNA was only detected in the B lymphocyte-enriched fraction (Fisher's exact test, p<0.05). To further demonstrate a differential accumulation of HAdV and EBV DNA in tonsillar lymphocyte subpopulations, the ratio of the viral DNA copy number from the T cell to the B cell-enriched subpopulation (T/B ratio) was calculated for each tonsil sample (Fig 5).

### HAdV typing in infected tonsils

To determine the HAdV types in the enriched tonsillar B and T lymphocyte fractions we used nested PCR to amplify the most variable regions of the HAdV hexon gene [36, 37] on the HAdV positive samples (Table 2). The PCR products were subjected to DNA sequencing and a phylogenetic tree was built based on the alignment of the nucleotide sequences of the nested PCR amplicons of HAdV and the relevant prototype sequences from GenBank (S2 Fig). The distribution of HAdV types in patient age groups and diagnosed tonsillar diseases is shown in Table 5. No significant difference was observed between the patient categories. However, by combining the patient groups irrespective of diagnosis we found that the majority of the older cohorts harbored HAdV-5 DNA [2 (100%) and 5 (83%) for adolescent and adults with tonsillar hypertrophy, 8 (100%) and 10 (77%) for adolescent and adults with chronic/recurrent tonsillitis], whereas young patients displayed a significantly fewer positive cases for HAdV-5 [20 (62%) and 2 (50%) for patients with tonsillar hypertrophy and chronic/recurrent tonsillitis, respectively; Fisher's exact test, p<0.05]. An analysis of the HAdV type distribution in the tonsillar lymphocyte subpopulations showed that HAdV-5 and HAdV-3 DNA were predominantly found in the T cell fractions (S3A Fig). No significant difference was found between the HAdV-5, HAdV-3 and HAdV-2 DNA copy numbers in patients' tonsils (S3B Fig).

**Table 5:** HAdV genotype distribution.

		HAdV positive patients					
Genotype	Study population	Tonsillar hypertrophy			Chronic/recurrent tonsillitis		
		1–9	10–19	≥ 20	1–9	10–19	≥ 20
**HAdV-1**	1/65 (1.5%)				1/4 (25%)		
**HAdV-2**	5/65 (8%)	5/32 (16%)					
**HAdV-3**	12/65 (18%)	7/32 (22%)		1/6 (17%)	1/4 (25%)		3/13 (23%)
**HAdV-5**	44/65 (68%)	17/32 (53%)	2/2 (100%)	5/6 (83%)	2/4 (50%)	8/8 (100%)	10/13 (77%)
**Mixed**^**a**^	2/65 (3%)	2/32 (6%)					
**Mixed**^**b**^	1/65 (1.5%)	1/32 (3%)					
**HAdV negative**	18/111 (16%)						
**Non-typable**	28/93 (30%)						
**Total**	**111**	**32**	**2**	**6**	**4**	**8**	**13**

^a^ HAdV-5 and HAdV-16

^b^ HAdV-5 and HAdV-3

## Discussion

Tonsil surgery, either tonsillectomy or tonsillotomy, is one of the most common surgical procedures performed on children. In Sweden, approximately 12,500 persons undergo tonsil surgery each year, of which approximately 50% are <15 years of age [39]. Viral etiology of tonsillar hypertrophy and chronic/recurrent tonsillitis in the Swedish population has not been established. The results from the present study establish that 93 (84%) of all 111 patients were positive for HAdV in the palatine tonsils. Our data is in line with two previous studies, where 72 to 79% of patient tonsils and adenoids were tested positive for HAdV DNA [19, 22]. In the present study clinically defined patient material was analysed, which allowed us to relate the HAdV and EBV prevalence in patients with tonsillar hypertrophy and chronic/recurrent tonsillitis.

By isolating the T and B lymphocyte-enriched fractions from the palatine tonsils we could characterise the prevalence of viral DNA in these two cell populations. Among the young patients with tonsillar hypertrophy, the T cell fraction contained significantly higher HAdV DNA copy number compared to the B lymphocyte-enriched fraction (Figs 3Aii and 5A), suggesting that HAdV preferentially resides in tonsillar T lymphocytes. Our data is in line with the study by Garnett *et al*. (2002), which showed that species C (HAdV-1, HAdV-2 and HAdV-5) DNA is enriched in CD3-expressing tonsillar T cells. Interestingly, compared to the patients with chronic/recurrent tonsillitis, the patients diagnosed with tonsillar hypertrophy demonstrated significantly higher number of tonsil samples where HAdV DNA was detected in both T and B cell-enriched fractions. Similar observation was made comparing the young patient group with adolescent and adult patient groups diagnosed with either tonsillar hypertrophy or chronic/recurrent tonsillitis (Fig 3).

Out of 93 positive patients, who had at least one of the tonsils positive for HAdV DNA by qPCR, 65 patients were also positive using the nested PCR method. We failed to type viral DNA from the remaining 28 patients. The reason for this failure is that higher viral loads are required for typing PCR compared to qPCR. Accordingly, these 28 patient samples showed low viral DNA copy number in qPCR. HAdV-5 was the most common type (68%) in the Swedish patients diagnosed with tonsillar hypertrophy and chronic/recurrent tonsillitis (Table 5). This finding was unexpected, given that HAdV-1 and HAdV-2 have been previously shown to have the similar prevalence as HAdV-5 in tonsillar tissues [9, 19]. However, it should be noted that in our study HAdV-5 was most prominent in the adult patient group (Table 5), whereas the other studies only analysed patients aged 1–19 years and 1–15 years, respectively [9, 19]. Hence, it is possible that pathogenic HAdV-5 infections in the tonsils are more common among the adult patients (≥ 20 years).

A remarkably high prevalence of HAdV-3 (18%) was detected in the analysed tonsil samples. Epidemiological profiles of HAdV infections have revealed that HAdV-3 is a common isolated type in patients with respiratory infections [19, 40–44]. Hence, our data from the Swedish patient cohort supports the high prevalence of HAdV-3 infections in human tonsils. HAdV-3 has been recently shown to establish persistent infections in the human T lymphocyte-based Jurkat cell line [45]. This observation together with our data suggests that in addition to species C (HAdV-1, HAdV-2 and HAdV-5) viruses [9, 22] also species B (HAdV-3) [45] virus might establish long-term infections in the tonsillar lymphocytes.

We also detected HAdV-16 (species B) in two different patients and to the best of our knowledge this is the first report of detecting the HAdV-16 infection in human tonsils. Unlike HAdV-5 and HAdV-3, HAdV-16 is rarely reported in the literature in association with clinical adenovirus infections [46]. Hence, further epidemiological and clinical studies are needed to evaluate the importance of HAdV-16 infection on adenovirus-associated disorders, including the tonsillar diseases.

Three patients had mixed infections with different types of HAdVs in their left and right tonsils, of which 2 (3%) were tested positive for HAdV-16 and HAdV-5, while 1 (1.5%) patient was infected with HAdV-3 and HAdV-5 in the right and left tonsils respectively (Table 5). Also 38 patients (41%) only had HAdV in one of their tonsils (Fig 1A). Except for five of these patients that had a DNA copy number higher than 100 in 10^6^ cells, most of the others had DNA copy numbers close to the qPCR detection limit. These findings could be due to the undetectable level of viral genomes in the other tonsil or it might be due to a single infection of the anatomically separated left and right tonsils.

In addition to HAdV, EBV prevalence in tonsillar hypertrophy and chronic/recurrent tonsillitis was analysed. Of 58 EBV positive patients, 36 (62%) and 22 (38%) were diagnosed with chronic/recurrent tonsillitis and tonsillar hypertrophy, respectively (Table 3). In the present study we detected EBV DNA in 32%, 65% and 68% of the patients in age groups of 1–9, 10–19 and 20 years, respectively (Table 3), which is in line with similar studies showing a prevalence of EBV DNA in tonsillar material ranging from 23 to 75% in young and adolescent patients (2–15 years) [47–50].

Our experimental approach allowed us to further analyse the HAdV and EBV co-infection in patient tonsil samples. Of 111 patients, 43 (39%) were tested positive for both HAdV and EBV (Table 4). We found no HAdV/EBV double-negative samples in the adult patient group (≥20 years) diagnosed with chronic/recurrent tonsillitis (Table 4). Since this proportion is unexpected given the observed frequency of HAdV (76% and 67% in females and males, respectively, Table 2) and EBV (71% and 56% in females and males, respectively, Table 3), our data suggest that the presence of HAdV and EBV independently correlate with chronic/recurrent tonsillitis. Although an EBV infection is associated with acute tonsillitis in infectious mononucleosis, any role of EBV in chronic/recurrent tonsillitis has to our knowledge not been described before. In this scenario, chronic/recurrent tonsillitis can be a consequence of local reactivation of latent EBV. Alternatively, although we find it highly unlikely, we cannot exclude that some of the patients might have had a subclinical disease during surgery. It should be noted that our interpretation is based on a relatively small patient cohort and that further studies are warranted to confirm this finding.

We also quantified the EBV DNA copy number in the T and B cell-enriched fractions from our palatine tonsil samples (Figs 4 and 5B). Consistent with previous results our data indicate that the B cell-enriched fraction obtained from the EBV positive tonsils contained higher levels of EBV DNA compared to the T cell fraction [27, 51]. It is known that following the primary infection in the oropharynx, EBV persists at different sites like palatine and pharyngeal tonsils, lymph nodes and peripheral blood. In persistently infected tonsils, the latent form of EBV is found in the isolated B cells, detected by the RT-PCR method [27, 51, 52]. Also, EBV latent proteins have been detected in tonsillar CD20^+^ B cells (82%), CD3^+^ T cells (7%) and intermediate lineage cells (11%) [27]. Therefore our analyses of palatine tonsils confirm that EBV preferably accumulates in the B cell-enriched population. However, in comparison to the tonsillar hypertrophy group, the patients diagnosed with chronic/recurrent tonsillitis showed significantly higher number of the tonsil samples, where EBV DNA was only detected in the B cell-enriched fraction (Fig 4).

We were unable to detect any HCMV DNA in the enriched tonsillar B and T lymphocyte populations, which in line with other studies [53–55] indicate that the presence of HCMV in tonsillar material is a rare event (0 to 5%). Since monocytes are believed to be the major site of HCMV latent infection in peripheral blood mononuclear cells, it might be possible to test the tonsillar macrophages for the presence of HCMV DNA.

Collectively, our results show that HAdV and EBV infections are associated with tonsillar diseases. We show that tonsillar T and B lymphocytes are the prominent targets for HAdV and EBV infections, respectively. Interestingly, in a significant proportion of the young patients (1–9 years) with tonsillar hypertrophy, HAdV was also detected in the tonsillar B cell-enriched fraction. This observation indicates that the acute HAdV infections might contribute to the development of tonsillar hypertrophy among the young patients.

## Supporting Information

S1 Fig HAdV DNA copy number in CD20^+^- and CD2^+^-immunosorted tonsillar lymphocytes. (A) MNCs from three HAdV positive tonsil samples (10R, 19L, 24L) were immunosorted with anti-CD2 and anti-CD20 antibodies using BD FACSaria III cell sorter as described in the supporting materials and methods section (S1 Text). FACS profiles and the purity (%) of the collected CD2^+^ T and CD20^+^ B cell fractions are shown. (B) HAdV DNA copy number (log10/10^6^ cells) in FACS-isolated B and T cell fractions.
(PDF)

S2 Fig Phylogenetic analysis of HAdV based on the partial hexon gene sequence. The phylogenetic tree was constructed by the Maximun Likelihood method and bootstrap values determined by 1000 replications in SeaView. Detected HAdVs belong to species B and C, while HAdV-5 is the most prevalent type. Annotated HAdV reference types are indicated (•).
(PDF)

S3 Fig Prevalence of HAdV types in infected tonsills. (A) Prevalence of HAdV types in the isolated tonsillar B and T cell-enriched fractions. Each data point indicates HAdV DNA copy number (log10/10^6^ cells) in tonsillar B and T cell-enriched fractions. HAdV types are shown on x-axis. The horizontal bar shows median value for each group. Dotted line represents the threshold below which virus DNA was undetectable. *p<0.05, **** p<0.0001, determined by Mann-Whitney U test. (B) Prevalence of HAdV types in single HAdV-infected tonsils. Each data point indicates the summarised HAdV DNA copy number (log10/10^6^ cells) in a single tonsil. The horizontal bar shows median value for each group. HAdV types are shown on x-axis.
(PDF)

S1 Text Supporting materials and methods. (DOCX)
