# Supplementary figures and images for: Distribution and Molecular Characterization of Human Adenovirus and Epstein-Barr Virus Infections in Tonsillar Lymphocytes Isolated from Patients Diagnosed with Tonsillar Diseases
Source: PLoS One. 2016 May 2;11(5):e0154814. doi: 10.1371/journal.pone.0154814 (PMC4852932; doi:10.1371/journal.pone.0154814)

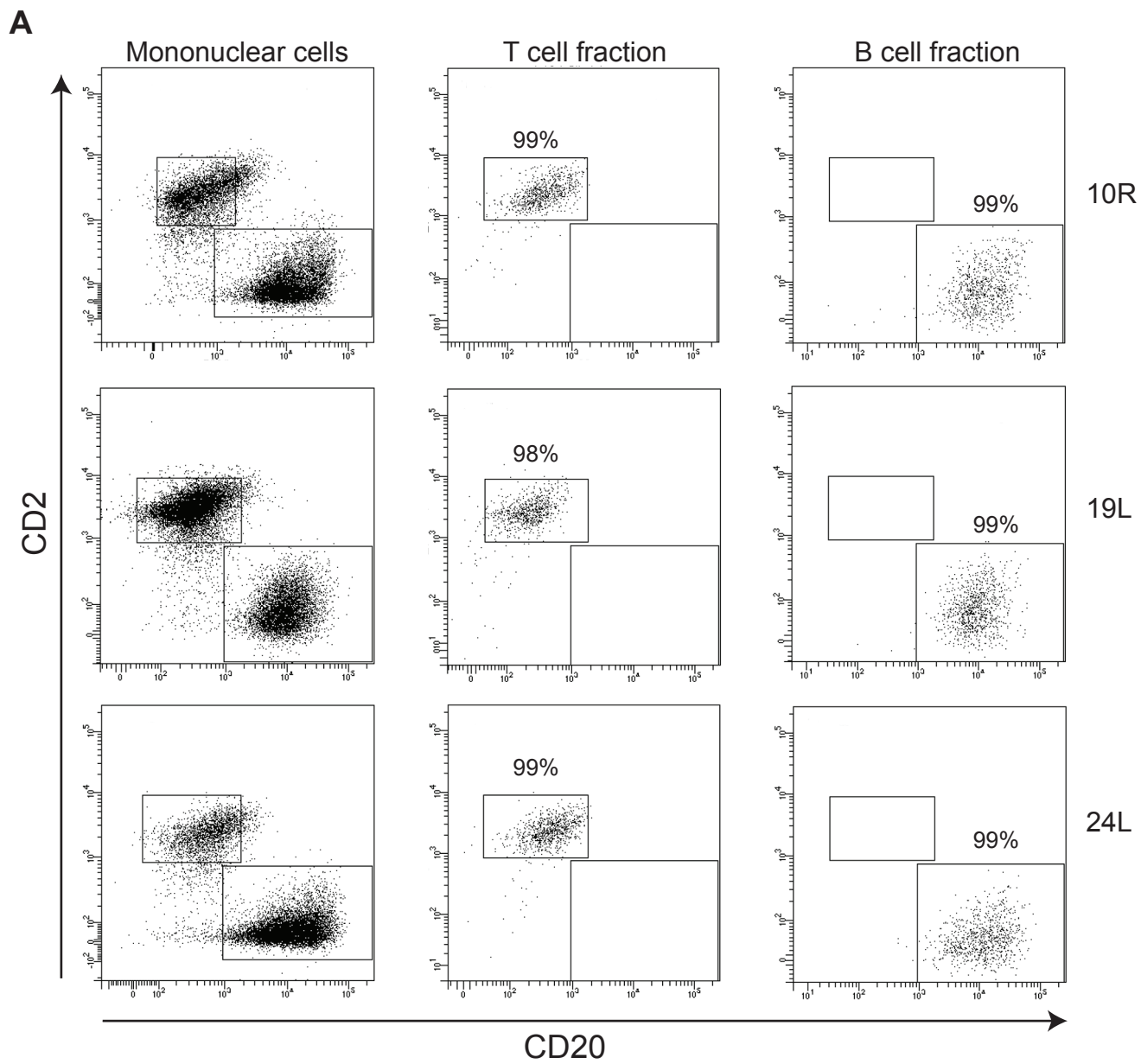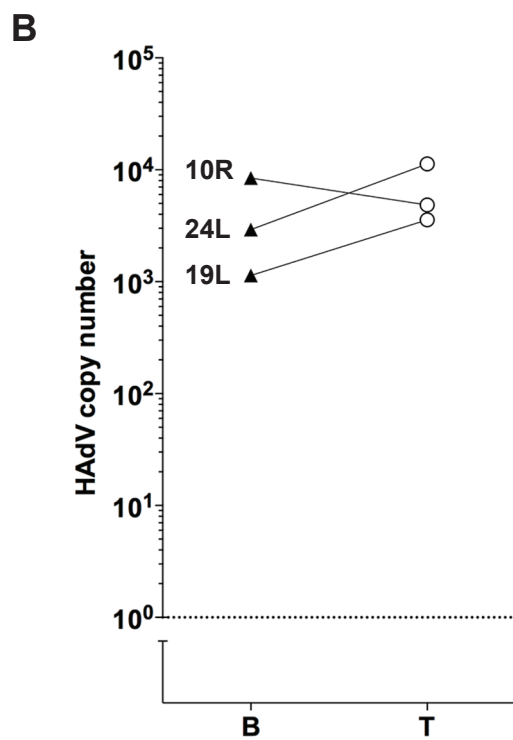

S1 Fig.

Supplement: S1 Fig — (A) MNCs from three HAdV positive tonsil samples (10R, 19L, 24L) were immunosorted with anti-CD2 and anti-CD20 antibodies using BD FACSaria III cell sorter as described in the supporting materials and methods section (S1 Text). FACS profiles and the purity (%) of the collected CD2+ T and CD20+ B cell fractions are shown. (B) HAdV DNA copy number (log10/106 cells) in FACS-isolated B and T cell fractions. (PDF) [file pone.0154814.s001.pdf]

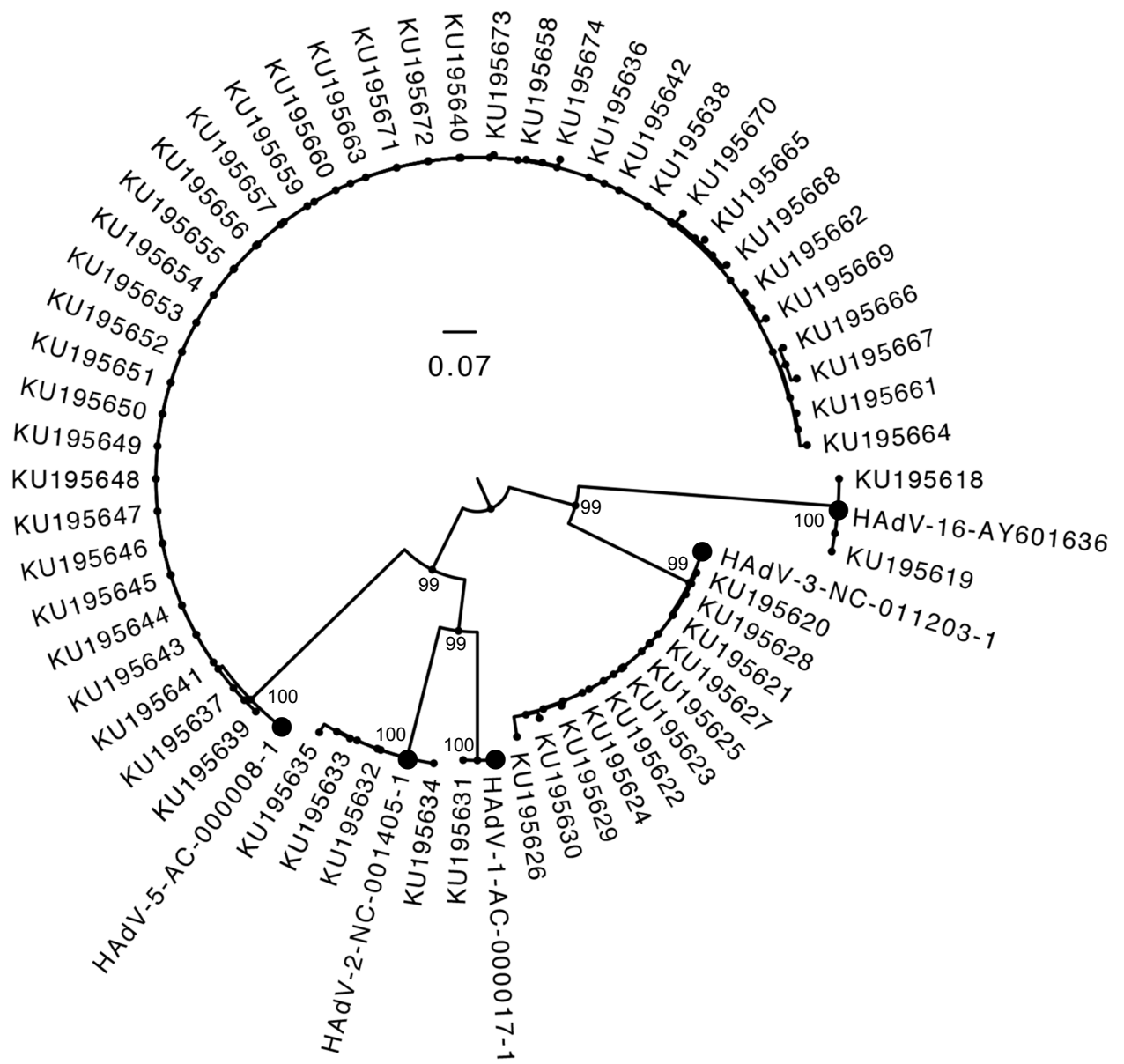

S2 Fig.

Supplement: S2 Fig — The phylogenetic tree was constructed by the Maximun Likelihood method and bootstrap values determined by 1000 replications in SeaView. Detected HAdVs belong to species B and C, while HAdV-5 is the most prevalent type. Annotated HAdV reference types are indicated (•). (PDF) [file pone.0154814.s002.pdf]

A

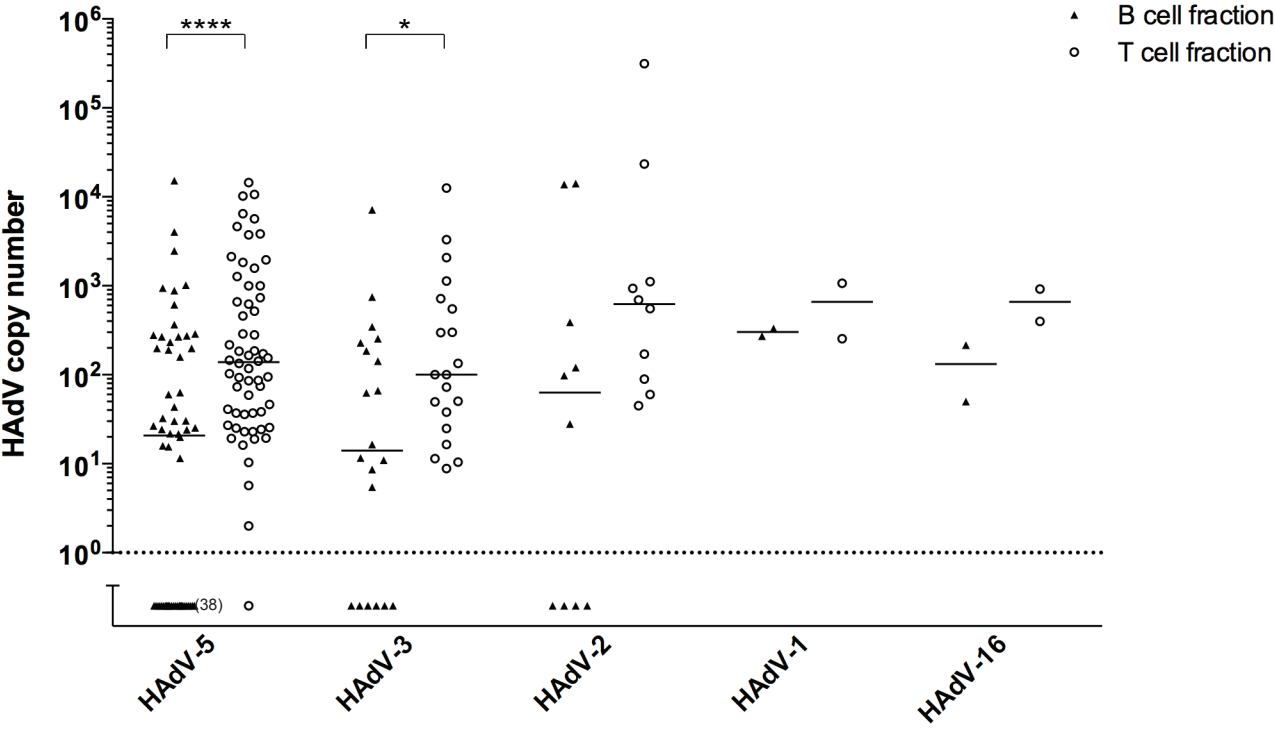

B

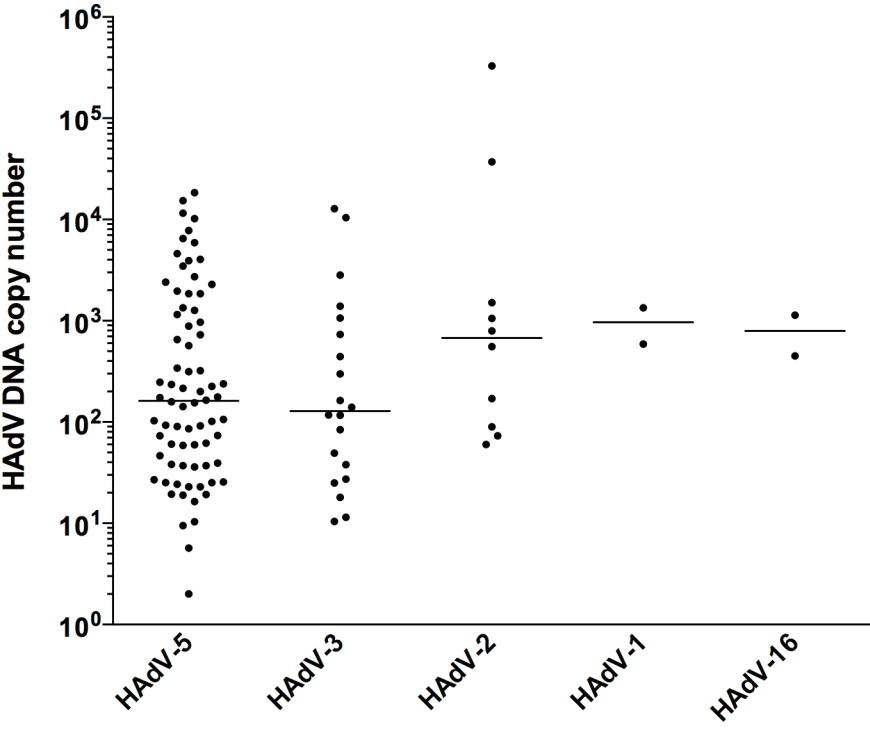

S3 Fig.

Supplement: S3 Fig — (A) Prevalence of HAdV types in the isolated tonsillar B and T cell-enriched fractions. Each data point indicates HAdV DNA copy number (log10/106 cells) in tonsillar B and T cell-enriched fractions. HAdV types are shown on x-axis. The horizontal bar shows median value for each group. Dotted line represents the threshold below which virus DNA was undetectable. *p<0.05, **** p<0.0001, determined by Mann-Whitney U test. (B) Prevalence of HAdV types in single HAdV-infected tonsils. Each data point indicates the summarised HAdV DNA copy number (log10/106 cells) in a single tonsil. The horizontal bar shows median value for each group. HAdV types are shown on x-axis. (PDF) [file pone.0154814.s003.pdf]
