# Supplementary material for: Distribution and Molecular Characterization of Human Adenovirus and Epstein-Barr Virus Infections in Tonsillar Lymphocytes Isolated from Patients Diagnosed with Tonsillar Diseases
Source: PLoS One. 2016 May 2;11(5):e0154814. doi: 10.1371/journal.pone.0154814 (PMC4852932; doi:10.1371/journal.pone.0154814)
Supplement: S1 Text — (DOCX) [file pone.0154814.s004.docx]

**Supporting information**

**Materials and methods**

**Phylogenetic analysis of HAdV species isolated from the present cohort**

The nucleotide sequences obtained from HAdV nested PCR amplicons, were aligned using the MAFFT algorithm [1] implemented in Geneious R7 (Biomatters, New Zealand). The nucleotide substitution model was determined in MEGA 6.0 [2] and maximum likelihood tree was constructed using PhyML [3] and bootstrapped 1000 times in SeaView [4]. Trees were projected using FigTree v1.4.2 [5]. Bootstrap values greater than 80% were considered significant.

**Purification of the tonsillar B and T lymphocytes by Fluorescence Activated Cell Sorting (FACS)**

Cryopreserved MNCs were thawed in a 37 °C water bath and immediately dispersed into 10 ml of pre-warmed Hanks balanced salt solution (HBSS, Gibco) supplemented with 5% fetal bovine serum (FBS, PAA), 10 mM Glutamine, 0.05 mg/ml Gentamicin and 1% Antibiotic-Antimycotic mix (Penicillin, Streptomycin and Amphotericin B, Gibco). The cells were spun down at 300 × g for 5 min and the resulting cell pellet was re-suspended in HBSS. Prior to the immunostaining, MNCs rested for 2 hours in supplemented HBSS medium at 37 °C and 5% CO_2_.

To obtain a single cell suspension for FACS staining, approximately 2-3 × 10^7^ MNCs were filtered through a 40 μM plastic cell strainer. The cells were washed and re-suspended in ice-cold PBSA (PBS containing 0.2% BSA) to reach the density of 10^7^ cells/ml. In order to block the Fc receptors, cells were incubated with 1 ml of PBS containing 10% heat inactivated human serum (Rockland Immunochemicals) for 1 min at room temperature. After washing in ice-cold PBSA, B and T lymphocyte surface antigens were stained with anti-CD20 and anti-CD2 monoclonal antibodies (BD Biosciences), respectively, as previously described [6]. The stained cells were sorted using BD FACSaria III  cell sorter (BD Biosciences). Subsequent FACS data analysis was performed using BD FACSDiva 8.0.1 software (BD Biosciences). An 85-m ceramic nozzle (BD Biosciences), a sheath pressure of 35-40 pounds per square inch (PSI) and an acquisition rate of 1000-2000 events per second were used as parameters for the cell sorting. The sorting speed was adjusted to ensure that the sorting efficiency was above 90%. The purity of the sorted fractions was checked after acquisition of at least 5 × 10^6^ cells. DNA was extracted from immunosorted tonsillar B and T lymphocyte fractions and the presence of HAdV DNA in these samples was detected by qPCR as previously described [6].

**References**

1. Katoh K, Asimenos G, Toh H. Multiple alignment of DNA sequences with MAFFT. Methods in molecular biology. 2009;537:39-64. doi: 10.1007/978-1-59745-251-9_3. PubMed PMID: 19378139.

2. Tamura K, Stecher G, Peterson D, Filipski A, Kumar S. MEGA6: Molecular Evolutionary Genetics Analysis version 6.0. Molecular biology and evolution. 2013;30(12):2725-9. doi: 10.1093/molbev/mst197. PubMed PMID: 24132122; PubMed Central PMCID: PMC3840312.

3. Guindon S, Delsuc F, Dufayard JF, Gascuel O. Estimating maximum likelihood phylogenies with PhyML. Methods in molecular biology. 2009;537:113-37. doi: 10.1007/978-1-59745-251-9_6. PubMed PMID: 19378142.

4. Gouy M, Guindon S, Gascuel O. SeaView version 4: A multiplatform graphical user interface for sequence alignment and phylogenetic tree building. Molecular biology and evolution. 2010;27(2):221-4. doi: 10.1093/molbev/msp259. PubMed PMID: 19854763.

5. Rambaut A. FigTree v1.4.2: Tree figure drawing tool. Available: <http://tree.bio.ed.ac.uk/software/figtree/>. Accessed November 2015. 2008.

6. Assadian F, Sandstrom K, Laurell G, Svensson C, Akusjarvi G, Punga T. Efficient Isolation Protocol for B and T Lymphocytes from Human Palatine Tonsils. Journal of visualized experiments : JoVE. 2015;(105). doi: 10.3791/53374. PubMed PMID: 26650582.
